# The 11th annual conference of The Australia & New Zealand Academy for Eating Disorders

**DOI:** 10.1186/2050-2974-1-S1-I1

**Published:** 2013-11-06

**Authors:** Jeremy Freeman

**Affiliations:** 1 PO Box 4154, Castlecrag, NSW, 2068, Autstralia

## 

The 11th annual conference of The Australia & New Zealand Academy for Eating Disorders was held on 23-24 August 2013 at the Mercure Pullman Hotel Melbourne. 71 Oral papers and 11 Posters were presented following review of the Scientific Committee chaired by Professor Susan Paxton and Dr Beth Shelton and are published in this supplement. Over 400 delegates attended the conference.

Keynote talks were by Dr Susie Orbach; ***The Politics of the body and the body politic*** and Dr Joe Proietto; ***Why is it so difficult to maintain weight loss: is this failure related to binge eating disorder and bulimia nervosa?*** The conference also involved two Plenaries, ***I-Tools & the internet*** (Amy Slater, Jenna Tregarthan and Jane Miskovic) and ***Interventions for the overweight client*** (Sue Byrne and Leah Brennan) as well as a case panel discussion featuring Susie Orbach, Anthea Fursland and Carolyn Costin.

Seven pre-conference workshops focussed on medical, nursing dietetic and psychological approaches to eating disorders. Susie Orbach’s workshop addressed the interpersonal history and psychology of the body. Jennifer Gaudiani presented insightful and highly rated workshops on medical issues at both introductory and advanced levels. Andrew Wallis and Linsey Atkins provided insight into Family-based Therapy clinical practice.

